# A Manual of Genito-Urinary and Venereal Diseases

**Published:** 1895-09

**Authors:** 


**A Manual of Genito-Urinary and Venereal Diseases,** by Bukk G. Carleton, M. D., with Venereal Diseases of the Eye, by Charles Deady, M. D., and the Vesical Calculus and External Urethrotomy, by Wm. Francis Homan, M. D. New York: Bœricke, Runyon & Ernesty, 1895. Price, $3.00.

We enter upon the criticism of this book by quoting the preface:

"It has been many years since a concise treatise on genito-urinary and venereal diseases, convenient for ready reference, has been offered for public consideration. The fact that many changes in the treatment have become necessary from a better understanding of the diseases themselves, the rapid advance in antiseptic and aseptic surgery, and a better general knowledge of Bacteriology, and its relations to cause and effect, pointing the way in many cases to their more rational treatment, to say nothing of the new remedies and operations advocated in the past few years, are sufficient reasons in our judgment for the presentation of this book."

Having now learned the author's motive for writing the book, we proceed to examine the text.

Here we find clear-cut, useful explanations of the various diseases; excellent descriptions of instruments, with admirable wood-cuts, and indications for homœopathic remedies. But rational medicine enters here also. Thus in gonorrhœa, especially in the female, germicides must be given to destroy the gonococcus of Neisser. "Alkalinity of the urine must be maintained to prevent and lessen the scalding as it passes over the inflamed labia." The external parts must be kept clean by frequent bathing with Calendula and Borax, dusted with Subnitrate of Bismuth or Oleate of Zinc; douches of Bichloride of Mercury and Chlorate of Potash; tampons of Glycerine, Boroglyceride, Subnitrate of Bismuth, etc.

With all this adjuvant treatment, there is but small chance for the action of the homœopathic remedy or of an intelligent recognition of the action of the homœopathic remedy if it should occur.

In speaking of the treatment of gonorrhœa in the male, lists of remedies are given, but no indications for them. On the other hand, prescriptions of more than one drug are given; as, for instance, in chordee a prescription of Antipyrin and Bromide of Potassium. Irritation of the neck of the bladder is treated with Extract of Hyoscyamus and Cannabis-indica, of each eight grains, mixed with Saccharum-album forty-eight grains, and made into twenty powders. These prescriptions are credited by the author to "traditional medicine." Nevertheless, there they stand for use, and in close contact with lists of homœopathic medicines given without any accompanying indications by which to select them for these same ailments. The urethra is to be treated with injections of Sulphate of Zinc and Aqueous extract of Hydrastis; Zinc Sulpho-carbolate and rose water; Zinc-permanganate and water; Boracic Acid, Aqueous Extract of Opium and Subacetate of Lead; Bismuth Salicylate, Boracic Acid, and Hydrogen-peroxide.

This is not desirable in a volume that is professedly devoted to homœopathic medicines, especially as all such prescriptions can be found in the books of the regular school of treatment. If this kind of treatment is in accordance with the authorized teachings of Homœopathy, then Homœopathy has no excuse for a separate existence as a system of medicine. Its books are superfluous additions to the library of the physician of universal education and method of practice, and its pharmacy a confusing triviality that ought to be abolished.

If it be said that experience demands this method of treatment with such a vicious disease as gonorrhœa, the answer can be made that the author of this criticism has treated many a case of gonorrhœa without these polypharmaceutic prescriptions, and he therefore knows whereof he speaks.

Instrumental treatment is carefully described and the instruments finely illustrated, especially in such conditions as stricture of the urethral canal.

Gonorrhœal rheumatism is described with care, and indications for homœopathic remedies given in detail. Gonorrhœal inflammation of the eye is treated with homœopathic remedies, the detailed symptomatology being given. But the eye is also treated with disinfecting solutions, such as Bichloride of Mercury, Boric Acid, and Chlorine Water. The pupil must be kept dilated with Atropine "to prevent iritic complication." The conjunctiva is to be painted with Nitrate of Silver, and so on.

Chancroids are treated with escharotics like Nitric and Sulphuric-acids, Acid Nitrate of Mercury, the actual cautery, and the knife. The indications for homœopathic remedies are absent. True chancres are not to be cauterized, but should be dusted with Iodoform, Aristol, Calomel, etc. Some homœopathic remedies are recommended but indications are not given.

Considerable space is devoted to syphilis and the descriptions are clear-cut and yet short. But the treatment is rational and does not show that the homœopathic method of selecting drugs, and administering them is of any service in the treatment of these diseases.

As a contribution to medical literature, this book is certainly an elegant production, and the fine printing, excellent paper, and attractive binding certainly add to its acceptability. But for enabling a student to select medicines on the homœopathic plan, to reach these disorders, or for inspiring him with any kind of trust in the efficacy of remedies thus selected, the work cannot, in the judgment of the writer, be considered a success.

